# Association between urine uranium and asthma prevalence

**DOI:** 10.3389/fpubh.2023.1326258

**Published:** 2024-01-08

**Authors:** Dongdong Huang, Saibin Wang

**Affiliations:** ^1^Department of Respiratory and Critical Care Medicine, The Fourth Affiliated Hospital, Zhejiang University School of Medicine, Yiwu, Zhejiang, China; ^2^Department of Pulmonary and Critical Care Medicine, Jinhua Municipal Central Hospital, Jinhua, China

**Keywords:** uranium, asthma, heavy metal, environmental pollution, NHANES

## Abstract

**Background:**

Previous studies showed that urine uranium (U) is associated with asthma prevalence in adults. However, the association between them among the general population is unclear. Therefore, this study aimed to explore this unclear association.

**Methods:**

The data of the participants were collected from the 2007–2016 National Health and Nutrition Examination Survey (NHANES) performed in the United States. Continuous variables with a skewed distribution were analyzed using Ln-transformation. The association between urine U and asthma prevalence was analyzed by multiple regression analysis, and the linear association between them was evaluated by smoothed curve fitting. The subgroup analysis was performed using the hierarchical multivariate regression analysis.

**Results:**

A total of 13,581 participants were included in our analysis. The multivariate regression analysis showed that LnU was independently and positively correlated with asthma prevalence in the general population (OR = 1.12; 95% CI: 1.04–1.20; *p* = 0.002). The subgroup analysis revealed that college graduate or above showed a stronger association between LnU and asthma prevalence (<9th grade: OR = 0.84; 95% CI: 0.61–1.14; 9–11th grade: OR = 1.23; 95% CI: 0.99–1.52; high school grade: OR = 1.00; 95% CI: 0.84–1.19; college: OR = 1.04; 95% CI: 0.91–1.19; ≥college graduate: OR = 1.32; 95% CI: 1.11–1.57; *P* for interaction = 0.0389).

**Conclusion:**

Our research suggested that urinary U levels are positively associated with asthma prevalence among the general population of the United States, and the association is especially strong among people with high levels of education.

## Highlights

- The data from NHANES, an extensive sample survey with a random sampling of the general population in the United States, were used as it has a good population representation.
- Urine uranium was positively correlated with asthma prevalence in the United States’ general population.
- Our research indicates that the educational level has a significant interaction effect on the association between urinary U levels and asthma prevalence in the general population. The correlation between U and asthma prevalence is stronger among individuals with higher levels of education.

## Introduction

Asthma is a chronic inflammatory disease of the airways, affecting approximately 300 million people worldwide. It has become a serious global health problem affecting all humankind, as revealed by the Global Initiative for Asthma (GINA) report in 2021 (1). It has been demonstrated through various investigations that asthma management remains inadequate in the United States, Canada, Europe, and Asia (2–4). In addition, Yaghoubi et al. estimated that the total economic burden of uncontrolled asthma among adolescents and adults in the United States will exceed $900 billion over the next 20 years (5).

The pathogenesis of asthma is relatively complicated. Asthma is related to early abnormal immune maturation, genomics, and environmental factors, and it is a heterogeneous disease (6). Environmental factors play a very important role in asthma prevalence. Indeed, an association between uranium (U) and asthma prevalence in adults was found in China and the United States (7–9). While the study conducted in China investigated the correlation between asthma in children and adults and heavy metal exposure, as indicated by urinary levels, it is essential to note that the data were derived from hospital-based case–control studies (7). Consequently, it is possible that some asymptomatic cases were not captured, as individuals who did not seek treatment at hospitals were not included in the analysis. In contrast, two separate cross-sectional studies conducted in the United States exclusively focused on adults and suggested a potential link between urinary U concentrations and asthma (8, 9). Notably, these studies did not encompass pediatric populations.

Exposure to harmful heavy metals such as lead (Pb), mercury (Hg), and arsenic (As) can cause a series of human diseases (10, 11). Among them, uranium (U) poisoning can cause serious health problems such as respiratory diseases, lung cancer (7–10, 12), renal toxicity (13), reproductive system toxicity (14), and immune system damage (15). The sources of U pollution include mining, military activities, nuclear facilities, groundwater, and phosphate fertilizers (16). Currently, only a few studies have reported the association between U exposure and adult asthma prevalence, and the epidemiological association between U exposure and asthma prevalence in the general population remains unclear (7–9). Because of the enormous harm of asthma to human health and the economic burden, a large sample of epidemiological data on the impact of environmental factors on asthma is necessary to carry out the primary prevention of asthma.

Therefore, this study was performed using data from the National Health and Nutrition Examination Survey (NHANES), which is a program of studies conducted to assess the health and nutritional status of adults and children in the United States, to investigate the association between urine U and asthma prevalence in the general population. The association between them in terms of age, gender, body mass index (BMI), economic level, race, education level, marital status, diabetes, hypertension, liver disease, smoking, alcohol consumption, and kidney function was also explored to evaluate the influence of these factors.

## Methods

### Study population

Our analysis was performed on NHANES data from 2007 to 2016. These data were obtained through a multi-level probability sampling of the nutrition and health status of the United States population, representative of the general population in the United States. We included participants aged 6–150 years in the 2007–2016 NHANES database. A total of 50,588 participants were considered in this analysis. However, participants who met the following exclusion criteria were removed: participants who were unsure whether they had asthma (*n* = 2,159); those without urine U values (*n* = 34,739); and pregnant women (*n* = 109). After these exclusions, a total of 13,581 participants were included in our study.

### Exposure variable

Urine U was the exposure variable in this study, detected by inductively coupled plasma mass spectrometry (ICP-MS). ICP-MS detects the U ion intensity, and from that, it elaborates on the concentration. The urine samples were collected, processed, and transported to the National Environmental Health Center, the Department of Environmental Health Laboratory Science, and the Centers for Disease Control and Prevention to be analyzed. A detailed description of the method used for urine U detection is available on the following website: https://wwwn.cdc.gov/Nchs/Nhanes/.

### Outcome variable

The outcome variable in this study was the self-reported prevalence of asthma. Participants were asked the question, “Has a doctor or other health professional ever told {you/SP} that {you have/s/he/SP has} asthma (az-ma)?”. The answer to this question was divided into “Yes” or “No.” The participants were split into the asthma and non-asthma groups according to participants’ self-reporting on whether they had asthma or not.

### Covariates

Due to the limited number of research literature on the association between U and asthma, underlying confounding variables have not been determined. However, given the large sample size of our study, we have also included clinically relevant variables that may have an impact (7, 8). In addition, since U may be metabolized through the liver and kidneys, variables related to liver and kidney function were also included as covariates.

The following covariates that can be considered potential confounders associated with urine U and asthma prevalence were collected according to previous studies and clinical considerations: age, gender, race, BMI, poverty-to-income ratio (PIR), education level, marital status, blood sugar (diabetes), blood pressure (hypertension), liver diseases, smoking, and alcohol consumption. The following blood parameters were also collected: total protein (TP), albumin, globulin, alanine aminotransferase (ALT), aspartate aminotransferase (AST), blood urea nitrogen (BUN), serum creatinine (Scr), total bilirubin (TB), serum uric acid (SUA), and the content of the following metals in the urine were also collected: barium (Ba), cadmium (Cd), cobalt (Co), cesium (Cs), molybdenum (Mo), Pb, stibium (Sb), thallium (Tl), tungsten (Tu), and Hg.

Age, gender, race, PIR, education level, and marital status were obtained from the demographic data of NHANES. Race was divided into five categories: Mexican American, non-Hispanic white, non-Hispanic Black, other Hispanic, and other races. The BMI of the study population was calculated using the following formula: weight (kg)/height (m^2^). Education level included the following levels: <9th grade, 9–11th grade, high school grade, college, and ≥ college graduate. Marital status was classified as follows: married, divorced, widowed, separated, living with a partner, and never married. Information regarding the presence of diabetes, hypertension, liver disease, and whether they have smoking and drinking habits was obtained from the questionnaire filled out by the participants. If the participant smoked at least 100 cigarettes during their life, this behavior was defined as smoking behavior. Alcohol use was defined as drinking at least 12 glasses of alcoholic beverages in the past 12 months. Moreover, the Chronic Kidney Disease Epidemiology Collaboration (CKD-EPI) equation was used to convert the serum creatinine of the participants into the glomerular filtration rate (GFR) to assess and adjust the influence of renal function on the association between urine U and asthma prevalence (17, 18). A GFR of <60 mL/min/1.73 m^2^ was defined as renal insufficiency (19–22).

### Statistical analysis

Statistical analysis was performed using the R software (The R Foundation^1^). The baseline characteristics of the study population were evaluated by the descriptive analysis. Since the levels of Ba, Cd, Co, Cs, Mo, Pb, Sb, Tl, Tu, Hg, and U in urine were characterized by a skewed distribution, the Ln-conversion was performed to improve the normality. Age was presented as median (Q1–Q3). Continuous variables were indicated as mean ± standard deviation (SD), while categorical variables were indicated by numbers (percentages). A comparison between the asthma and non-asthma groups was performed using the unpaired *t*-test or Mann–Whitney *U*-test, Pearson’s chi-squared tests, or Fisher’s exact, as appropriate. The association between urine U and asthma prevalence was assessed by a multiple regression model.

Two adjustment models were used to explore the association between their associations. The covariates that contribute to the odds ratio change more significantly than 10%, such as age, gender, race, and BMI, were included as the adjustment variables for the adjusted model I. We performed multiple regression analysis to estimate the independent relationship between urine U and asthma prevalence, with an adjustment for potential confounders. The potential confounding variables in this study have been referred to in the literature related to U and asthma, as well as variables that may have an impact on the association between asthma and U through univariate statistical analysis (7–9). The adjusted model II included the variables in model I and the ones in which the *p*-value of the covariate to the dependent variable was less than 0.1, such as age, gender, race, BMI, education level, marital status, PIR, diabetes, hypertension, alcohol use, BUN, TB, TP, globulin, LnBa, LnCd, LnCo, LnCs, LnPb, LnSb, LnTl, LnTu, and LnHg. LnU was transformed into a quartile categorical variable, and the *p*-value for the trend was calculated to verify the possibility of non-linearity between LnU as a continuous variable and asthma prevalence.

Furthermore, the correlation graph between urine U and asthma prevalence was shown using smoothed curve fitting (penalty spline method). Multiple imputation analysis was used to assess whether the missing values of the covariates were the cause of bias in the results (23). Hierarchical multivariate regression analysis was used for subgroup analysis. A *p*-value of <0.05 was considered statistically significant.

## Results

### Baseline characteristics of the subjects

A total of 13,581 participants from the NHANES data from 2007 to 2016 were included in our analysis according to the inclusion and exclusion criteria of this study. The baseline characteristics of the study population are listed in Table 1. The participants in the asthma and non-asthma groups were 2,081 and 11,500, respectively. The median age in the asthma and non-asthma groups was 36.0 (15–57) and 29.0 (14–52) years, respectively (*p* < 0.001). The mean LnU of the asthma and non-asthma groups was −5.1 ± 1.0 and − 5.0 ± 1.0 ug/L, respectively (*p* < 0.001). In addition, the asthma and non-asthma groups showed a statistically significant difference in BMI, PIR, race, education level, marital status, diabetes, hypertension, liver disease, smoking, alcohol consumption, TP, albumin, globulin, BUN, TB, GFR, LnBa, LnCd, LnCo, LnPb, LnSb, LnTl, LnTu, and LnHg (all *p* < 0.05).

**Table 1:** Baseline characteristics of the study population in NHANES during the period 2007–2016.

Variables	Non-asthma (*n* = 11,500)	Asthma (*n* = 2081)	*p*-value
Age (years), Median (Q1–Q3)	36.0 (15–57)	29.0 (14–52)	<0.001
Gender (n), %			0.056
Man	5,816 (50.6%)	1,005 (48.3%)	
Woman	5,684 (49.4%)	1,076 (51.7%)	
BMI (Kg/m^2^), Mean ± SD	26.1 ± 7.3	27.5 ± 8.6	<0.001
PIR (Ratio), Mean ± SD	2.3 ± 1.6	2.2 ± 1.6	<0.001
Race (n), %			<0.001
Mexican American	2,210 (19.2%)	247 (11.9%)	
Other Hispanic	1,259 (10.9%)	238 (11.4%)	
Non-Hispanic white	4,186 (36.4%)	830 (39.9%)	
Non-Hispanic Black	2,485 (21.6%)	539 (25.9%)	
Other Races	1,360 (11.8%)	227 (10.9%)	
Education (n), %			<0.001
<9th Grade	958 (8.3%)	94 (4.5%)	
9–11th Grade	1,127 (9.8%)	182 (8.7%)	
High School Grade	1742 (15.1%)	296 (14.2%)	
College	2,173 (18.9%)	443 (21.3%)	
≥College Graduate	1791 (15.6%)	269 (12.9%)	
Do not know	3,709 (32.3%)	797 (38.3%)	
Marital status, N (%)			<0.001
Married	4,119 (35.8%)	556 (26.7%)	
Widowed	625 (5.4%)	92 (4.4%)	
Divorced	782 (6.8%)	180 (8.6%)	
Separated	248 (2.2%)	43 (2.1%)	
Never married	1,371 (11.9%)	306 (14.7%)	
Living with partner	648 (5.6%)	107 (5.1%)	
Do not know	3,707 (32.2%)	797 (38.3%)	
Diabetes, N (%)			<0.001
Yes	960 (8.3%)	230 (11.1%)	
No	10,348 (90.0%)	1812 (87.1%)	
Border line	188 (1.6%)	37 (1.8%)	
Do not know	4 (0.0%)	2 (0.1%)	
Hypertension, N (%)			<0.001
Yes	2,743 (23.9%)	554 (26.6%)	
No	5,861 (51.0%)	903 (43.4%)	
Do not know	2,896 (25.2%)	624 (30.0%)	
Liver Disease, N (%)			<0.001
Yes	278 (2.4%)	82 (3.9%)	
No	7,502 (65.2%)	1,199 (57.6%)	
Do not know	3,720 (32.3%)	800 (38.4%)	
Smoking, N (%)			<0.001
Yes	3,401 (29.6%)	682 (32.8%)	
No	4,554 (39.6%)	636 (30.6%)	
Do not know	3,545 (30.8%)	763 (36.7%)	
Alcohol use, N (%)			<0.001
Yes	5,124 (44.6%)	920 (44.2%)	
No	2,138 (18.6%)	324 (15.6%)	
Do not know	4,238 (36.9%)	837 (40.2%)	
Blood test, Mean ± SD			
TP (g/dL)	7.2 ± 0.5	7.1 ± 0.5	<0.001
Albumin (g/dL)	4.3 ± 0.3	4.3 ± 0.3	<0.001
Globulin (g/dL)	2.9 ± 0.5	2.9 ± 0.5	0.049
ALT (U/L)	24.6 ± 18.4	24.2 ± 15.9	0.47
AST (U/L)	25.7 ± 16.1	25.5 ± 12.9	0.603
BUN (mg/dL)	13.0 ± 5.5	12.5 ± 5.4	<0.001
Scr (mg/dL)	0.9 ± 0.3	0.9 ± 0.3	0.361
TB (mg/dL)	0.7 ± 0.3	0.7 ± 0.3	<0.001
SUA (mg/dL)	5.4 ± 1.4	5.4 ± 1.4	0.141
GFR (mL/min/1.73 m2)	98.3 ± 26.9	100.6 ± 27.3	0.002
Urine metals, Mean ± SD			
LnBa (μg/L)	0.2 ± 1.0	0.2 ± 1.0	0.032
LnCd (μg/L)	−1.9 ± 1.1	−2.0 ± 1.1	0.008
LnCo (μg/L)	−1.0 ± 0.8	−0.9 ± 0.8	<0.001
LnCs (μg/L)	1.4 ± 0.7	1.4 ± 0.7	0.852
LnMo (μg/L)	3.8 ± 0.9	3.8 ± 0.9	0.112
LnPb (μg/L)	−0.9 ± 0.9	−1.0 ± 0.9	0.002
LnSb (μg/L)	−2.9 ± 0.8	−2.8 ± 0.8	0.003
LnTl (μg/L)	−1.9 ± 0.7	−1.9 ± 0.7	0.039
LnTu (μg/L)	−2.5 ± 1.1	−2.4 ± 1.1	<0.001
LnHg (ng/mL)	−1.2 ± 1.1	−1.3 ± 1.0	0.039
LnU (ug/L)	−5.1 ± 1.0	−5.0 ± 1.0	<0.001

NHANES, National Health and Nutrition Examination Survey; SD, standard deviation; BMI, body mass index; PIR, poverty to income ratio; TP, total protein; ALT, alanine aminotransferase; AST, aspartate aminotransferase; BUN, blood urea nitrogen; Scr, serum creatinine; TB, total bilirubin; SUA, serum uric acid; GFR, glomerular filtration rate; Ba, barium; Cd, cadmium; Co, cobalt; Cs, cesium; Mo, molybdenum; Pb, lead; Sb, stibium; Tl, thallium; Tu, tungsten; Hg, mercury; U, uranium.

### Association between urinary U and asthma prevalence

**Figure 1:**
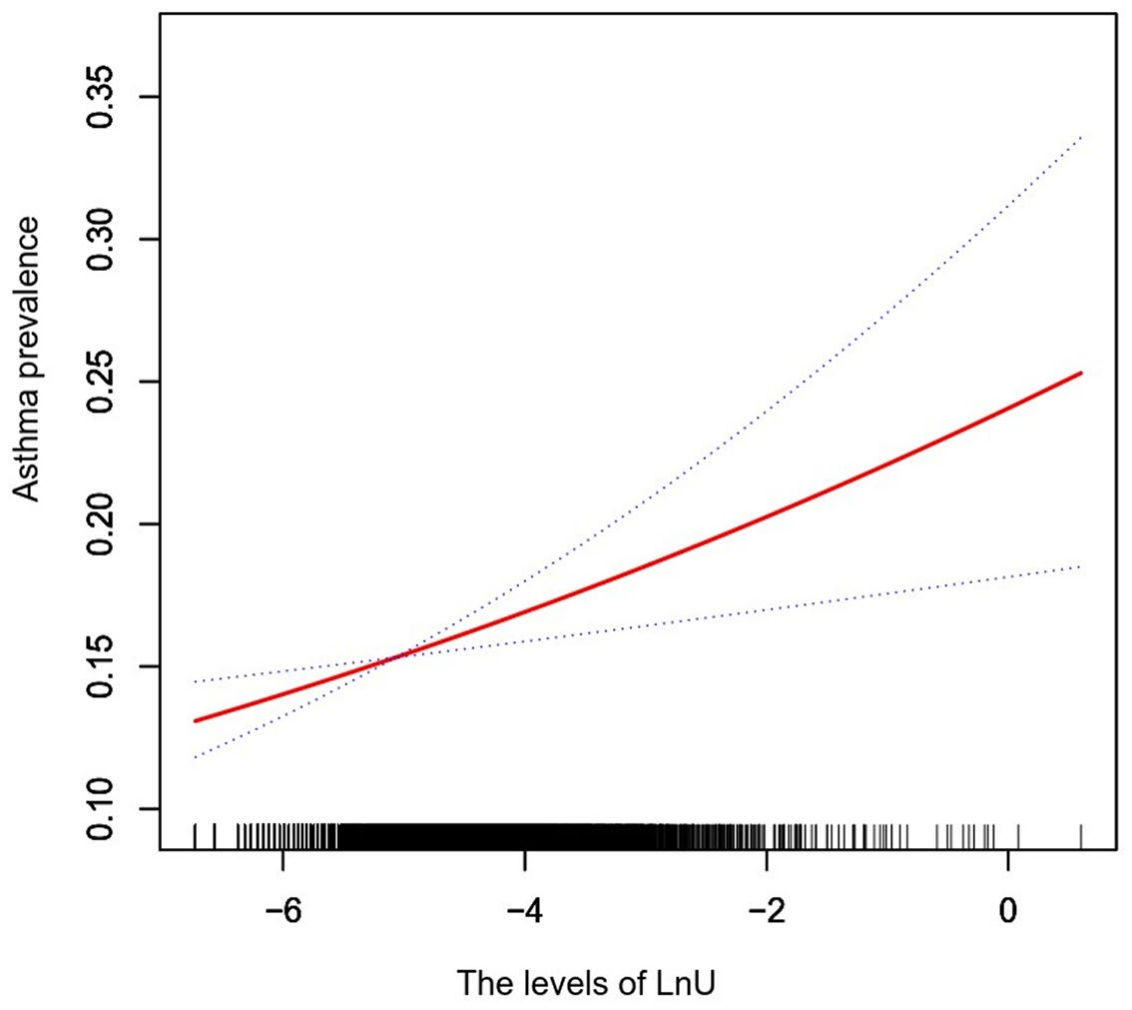
Smoothed curve fitting shows the association between urine LnU and asthma prevalence after adjusting for confounders (age, gender, race, BMI, education, marital status, PIR, diabetes, hypertension, alcohol use, BUN, TB, TP, globulin, LnBa, LnCd, LnCo, LnCs, LnPb, LnSb, LnTl, LnTu, and LnHg). The area between the two dashed lines indicates a 95% confidence interval. U, uranium; BMI, body mass index; PIR, poverty to income ratio; BUN, blood urea nitrogen; TB, total bilirubin; TP, total protein; Ba, barium; Cd, cadmium; Co, cobalt; Cs, cesium; Pb, lead; Sb, stibium; Tl, thallium; Tu, tungsten; Hg, mercury.

The association between urine U and asthma prevalence assessed by the logistic regression model is shown in Table 2. The continuous variable urine U was associated with asthma prevalence in the non-adjusted model (OR = 1.09; 95% CI: 1.04–1.14; *p* = 0.0003). Urine U was still associated with asthma prevalence after the adjustment of model I (OR = 1.10; 95% CI: 1.05–1.15; *p* < 0.0001) and model II (OR = 1.12; 95% CI: 1.04–1.20; *p* = 0.002) for different confounding factors. Moreover, participants in the second, third, and highest LnU quartile showed a statistically significantly higher asthma prevalence in the non-adjusted model (*P* for trend = 0.0007), adjustment model I (*P* for trend = 0.0002), and adjustment model II (*P* for trend = 0.0104) compared with the lowest-quantile LnU after converting LnU from a constant variable to a categorical variable (quartile). *P* for trends for all models was significant and consistent with the *p*-value of LnU as a continuous variable, thus suggesting a linear association between LnU and asthma prevalence. Further analysis with the smoothed curve fitting confirmed the linear association between urine LnU and asthma prevalence after the adjustment for confounding factors (adjustment model II) (Figure 1).

**Table 2:** Association between LnU and asthma prevalence in NHANES during the period 2007–2016.

	Asthma prevalence OR (95% CI) *p*-value		
	Non-adjusted	Adjusted Model I	Adjusted Model II
LnU	1.09 (1.04, 1.14) 0.0003	1.10 (1.05, 1.15) <0.0001	1.12 (1.04, 1.20) 0.0020
Quartiles			
LnU Q1	Ref. (1)	Ref. (1)	Ref. (1)
LnU Q2	1.19 (1.04, 1.36) 0.0137	1.18 (1.03, 1.35) 0.0209	1.31 (1.09, 1.56) 0.0033
LnU Q3	1.24 (1.09, 1.42) 0.0016	1.24 (1.08, 1.43) 0.0020	1.24 (1.03, 1.50) 0.0250
LnU Q4	1.26 (1.10, 1.45) 0.0007	1.30 (1.13, 1.49) 0.0002	1.38 (1.12, 1.69) 0.0022
*P* for trend	0.0007	0.0002	0.0104
Adjusted model I: age, gender, race, and BMI			
Adjusted model II: age, gender, race, BMI, education, marital status, PIR, diabetes, hypertension, alcohol use, BUN, TB, TP, globulin, LnBa, LnCd, LnCo, LnCs, LnPb, LnSb, LnTl, LnTu, and LnHg			

U, uranium; NHANES, National Health and Nutrition Examination Survey; OR, odds ratio; CI, confidence interval; BMI, body mass index; PIR, poverty to income ratio; BUN, blood urea nitrogen; TB, total bilirubin; TP, total protein; Ba, barium; Cd, cadmium; Co, cobalt; Cs, cesium; Pb, lead; Sb, stibium; Tl, thallium; Tu, tungsten; Hg, mercury.

**Figure 2:**
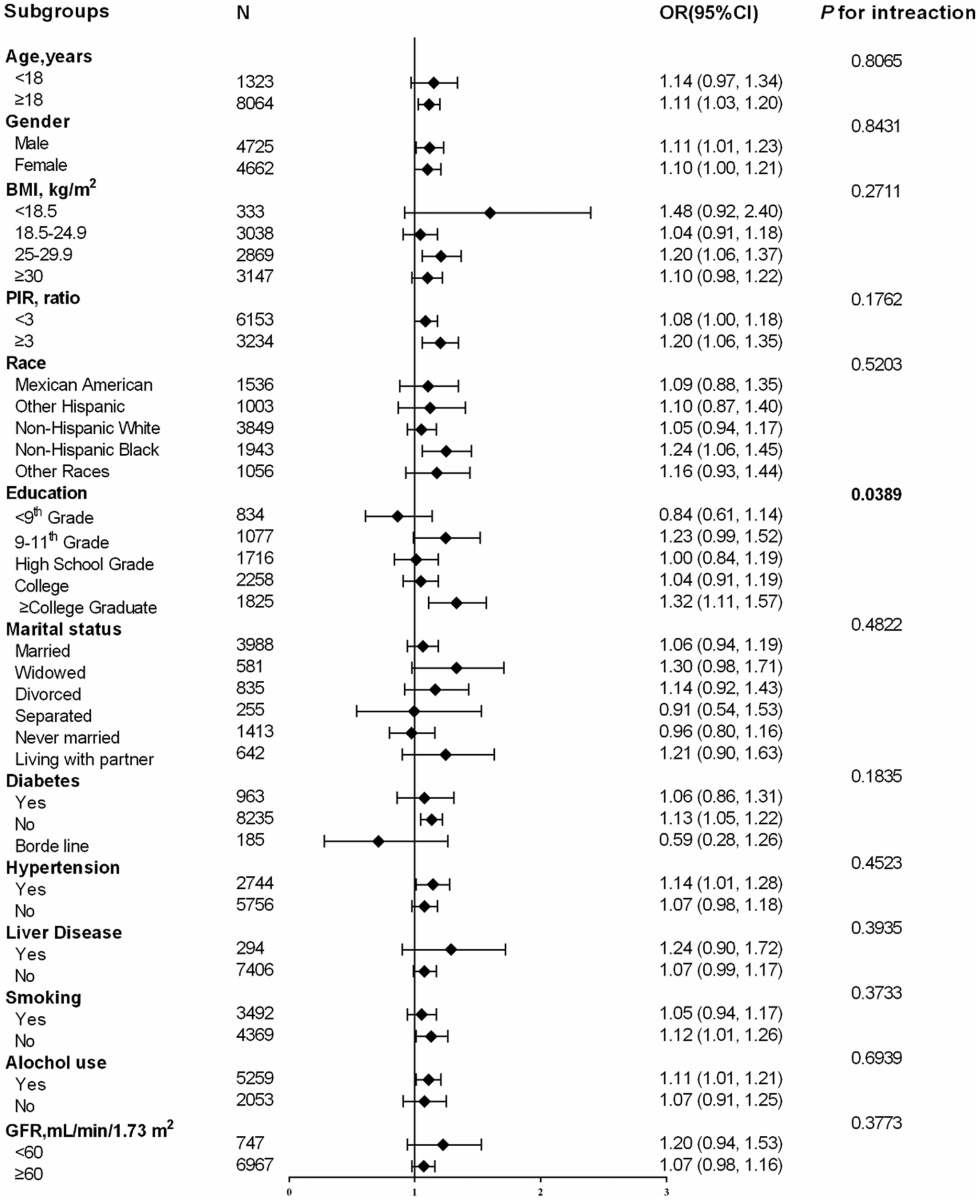
Subgroup analysis of the influence of LnU on asthma prevalence. OR, odds ratio; CI, confidence interval; BMI, body mass index; PIR, poverty to income ratio; GFR, glomerular filtration rate.

The effect of other covariates on the relationship between LnU and asthma prevalence was also evaluated in the subgroup analysis (Figure 2). The subgroup analysis revealed that college graduates or above had the strongest association between LnU and asthma prevalence (<9th grade: OR = 0.84; 95% CI: 0.61–1.14; 9–11th grade: OR = 1.23; 95% CI: 0.99–1.52; high school grade: OR = 1.00; 95% CI: 0.84–1.19; college: OR = 1.04; 95% CI: 0.91–1.19; ≥ college graduate: OR = 1.32; 95% CI: 1.11–1.57; P for interaction = 0.0389). The association between urine LnU and asthma prevalence was consistent in the following subgroups: age, gender, BMI, PIR, race, marital status, diabetes, hypertension, liver disease, smoking, alcohol use, and GFR (*P* for the interaction of all covariates >0.05). The results of the imputed data set and the complete data were consistent.

## Discussion

Our results demonstrated that the urine U level was positively correlated with asthma prevalence in the whole population of the US, and this association was particularly significant in people with an education level ≥ college graduate.

The bioavailability of U compounds in the human gastrointestinal tract is estimated between <0.1 and 6% (24). The kidney is the main excretory organ of U compounds (25). Since the urine U level is correlated with U exposure, urine U was used as a sign of U exposure in this study (26, 27).

A case–control study in a Chinese adult population (sample size 1:1 matched, 551 patients in both the asthma group and the control group) as well as an analysis considering 1,857 American adults in the NHANES 2007–2008 survey suggested that asthma prevalence was positively correlated with the level of U in the urine (7–9). Since then, this conclusion has been confirmed by Li, X et al., who obtained consistent results after considering 3,425 American adults aged 20–59 years (2011–2014) (8, 9). In this study, a sample size of 13,581 of the American general population was considered as the study population (NHANES 2007–2016), and their age was ≥6 years old, which basically represents the general population in the United States. The results of this study not only confirmed the association between urinary U levels and the incidence of asthma in adults but also were the first to reveal that asthma prevalence in adolescents and children (<18 years) positively correlated with urinary U levels. Our research has good clinical significance and represents a good reference value considering the high prevalence of asthma in adolescents and children, further revealing that this association cannot be ignored. In addition, our study also discovered that the education level of the general population had an interactive effect on the relationship between urinary U level and asthma prevalence since this association was stronger in people with higher levels of education. This suggests that their urinary U levels (U exposure) need more stringent management to reduce asthma prevalence in this population group. The pathogenesis of asthma is a complex and incompletely precise process involving multiple cellular components (28–30). Asthma caused by heavy metals is currently explained by the involvement of oxidative stress or airway inflammation (7). For example, immunocompetent cells are induced by heavy metals to produce oxidants (31), which can cause airway hyper-responsiveness (32), cause airway spasm and contraction (33), and increase airway mucus secretion (34). Additionally, Cr and Cd can induce airway inflammation, cause airway obstruction, and cause airway hyperresponsiveness (35, 36). Despite the above evidence, no research on the mechanism regulating the association between U and asthma prevalence is available. The potential mechanisms or pathways by which U exposure may lead to asthma are likely multifactorial. Several possible mechanisms include immune response, oxidative stress, neural regulation, and changes in gene expression. First, immune response may play a role while U can act as an immune stimulant, causing an overreaction of the immune system and leading to inflammation and respiratory stenosis. This response involves the activation of various immune cells, such as T and B lymphocytes, which release inflammatory mediators upon exposure to urine U, ultimately leading to respiratory inflammation and asthma. Second, oxidative stress may contribute to this process, as U exposure can cause cellular damage through reactive oxygen species molecules. Oxidative stress can result in respiratory epithelial cell damage and inflammation, thus promoting the development of asthma. Third, U exposure may affect the regulation of the nervous system, resulting in respiratory muscle contraction and airway narrowing. This neuroregulatory effect could involve neurotransmitters, including acetylcholine and norepinephrine, which may be altered after exposure to U, leading to respiratory muscle contraction and asthma symptoms. Finally, changes in gene expression may also play a role, as exposure to U can alter gene expression, affecting cell function and signal transduction. These changes can involve asthma-related genes, such as those involved in immune response, inflammation, and cellular signaling. Importantly, these mechanisms are interrelated and can work together to increase the risk of asthma in individuals exposed to U. However, further research is needed to confirm these mechanisms and gain a deeper understanding of the relationship between urinary U exposure and asthma. Thus, further longitudinal studies between U and asthma are necessary to discover the potential mechanism.

O’Conor et al. proposed that individuals with higher levels of education may have greater awareness regarding asthma compared to those with lower levels of education, and this factor may be more beneficial in the management of asthma (37). Our study revealed that the association between urine U and asthma prevalence in college graduates is more evident. First, this could be attributed to the fact that individuals with higher levels of education are more likely to prioritize their own health, thus enabling doctors or researchers to gather information regarding their asthma prevalence. Second, a potential indirect association between education levels in the United States and exposure to radioactive materials may exist. It is plausible that individuals with higher educational attainment are more likely to encounter high levels of U exposure due to their involvement in occupations or residential settings associated with radioactive materials. However, it is essential to note that this correlation does not imply a causal relationship. The impact of U exposure is highly individualized and influenced by factors including the dose, duration, and exposure method. Hence, it is necessary to monitor urinary U levels in the general population, especially in the population with a high level of education. In addition, further research is needed to explore the correlation between the level of education in the United States.

The data from NHANES, an extensive sample survey with a random sampling of the general population in the U.S., were used in this study since it has a good population representation. The multiple regression analysis was used to explore the independent relationship between urine U and asthma prevalence after adjusting for potential confounding factors. Despite this strength, this study also has some limitations. The simultaneous occurrence of U exposure and asthma cannot be considered a causal relationship between them since the design of this study is cross-sectional. A urine sample per participant was used to detect the urine U concentration, potentially causing measurement errors due to individual variability in short-term U excretion. Therefore, multiple or 24-h urine samples should be used to reduce measurement errors. Self-reported asthma history may have recall bias.

In summary, our research findings can serve as an important basis for public health promotion, a scientific basis for formulating environmental protection policies, and a reference for occupational health regulation and provide new ideas and directions for medical research.

## Conclusion

The results of our study suggest that there is a positive association between urinary U levels and asthma prevalence in the general population of the United States, particularly among individuals with higher levels of education. This association may be attributed to the fact that U is a toxic heavy metal, and long-term exposure to it may have detrimental effects on health, including the development of chronic respiratory diseases such as asthma. Therefore, this finding highlights the need for increased attention to the issue of U pollution in the environment. To mitigate the potential health risks associated with U exposure, government agencies should strengthen their monitoring and regulation of radioactive substances, reduce U levels in the environment, monitor urinary U concentrations, and implement measures to eliminate U pollution. Additionally, further research is needed to develop effective treatment methods for asthma and to better understand the impacts of exposure to radioactive substances.

## Data availability statement

The original contributions presented in the study are included in the article/supplementary material, further inquiries can be directed to the corresponding author.

## Ethics statement

The studies involving humans were approved by the research ethics review board of the National Center for Health Statistics. The studies were conducted in accordance with the local legislation and institutional requirements. Written informed consent for participation in this study was provided by the participants’ legal guardians/next of kin.

## Author contributions

DH: Writing – original draft, Writing – review & editing. SW: Writing – original draft, Writing – review & editing.

## Footnotes

^1^ https://www.r-project.org

## Glossary

U: Uranium
NHANES: National Health and Nutrition Examination Survey
OR: Odds ratio
CI: Confidence interval
GINA: Global Initiative for Asthma
Pb: Lead
Hg: Mercury
As: Arsenic
BMI: Body mass index
ICP-MS: Inductively coupled plasma mass spectrometry
PIR: Poverty to income ratio
TP: Total protein
ALT: Alanine aminotransferase
AST: Aspartate aminotransferase
BUN: Blood urea nitrogen
Scr: Serum creatinine
TB: Total bilirubin
SUA: Serum uric acid
Ba: Barium
Cd: Cadmium
Co: Cobalt
Cs: Cesium
Mo: Molybdenum
Sb: Antimony
Tl: Thallium
Tu: Tungsten
CKD-EPI: Chronic Kidney Disease Epidemiology Collaboration
GFR: Glomerular filtration rate
